# Development of novel noninvasive prenatal testing protocol for whole autosomal recessive disease using picodroplet digital PCR

**DOI:** 10.1038/srep37153

**Published:** 2016-12-07

**Authors:** Mun Young Chang, Ah Reum Kim, Min Young Kim, Soyoung Kim, Jinsun Yoon, Jae Joon Han, Soyeon Ahn, Changsoo Kang, Byung Yoon Choi

**Affiliations:** 1 Department of Otorhinolaryngology-Head and Neck Surgery, Chung-Ang University College of Medicine, 102 Heukseok-ro, Dongjak-gu, Seoul, 06973, Republic of Korea; 2 Department of Otorhinolaryngology, Seoul National University Hospital, Seoul national University College of Medicine, 101 Daehak-ro, Jongno-gu, Seoul 03080, Republic of Korea; 3 Department of Otorhinolaryngology, Seoul National University Bundang Hospital, 82 Gumi-ro 173 beon-gil, Bundang-gu, Seongnam, 13620, Republic of Korea; 4 LAS Inc., 16 Arayuk-ro, Gimpo, 10136, Republic of Korea; 5 Bio-Medical Science Co., Ltd., BMS Bldg., 22 Yeoksam-ro 7-gil, Gangnam-gu, Seoul 06244, Republic of Korea; 6 Medical Research Collaborating Center, Seoul National University Bundang Hospital, 82 Gumi-ro 173 beon-gil, Bundang-gu, Seongnam, 13620, Republic of Korea; 7 Department of Biology and Research Institute of Basic Sciences, College of Natural Sciences, Sungshin Women’s University, Dongseon-dong 3(sam)-ga, Seongbuk-gu, Seoul, 01133, Republic of Korea; 8 Wide River Institute of Immunology, Seoul National University College of Medicine, 101 Dabyeonbat-gil, Hwachon-myeon, Hongcheon, 25159, Republic of Korea

## Abstract

We developed a protocol of noninvasive prenatal testing (NIPT), employing a higher-resolution picodroplet digital PCR, to detect genetic imbalance in maternal plasma DNA (mpDNA) caused by cell-free fetal DNA (cffDNA). In the present study, this approach was applied to four families with autosomal recessive (AR) congenital sensorineural hearing loss. First, a fraction of the fetal DNA in mpDNA was calculated. Then, we made artificial DNA mixtures (positive and negative controls) to simulate mpDNA containing the fraction of cffDNA with or without mutations. Next, a fraction of mutant cluster signals over the total signals was measured from mpDNA, positive controls, and negative controls. We determined whether fetal DNA carried any paternal or maternal mutations by calculating and comparing the sum of the log-likelihood of the study samples. Of the four families, we made a successful prediction of the complete fetal genotype in two cases where a distinct cluster was identified for each genotype and the fraction of cffDNA in mpDNA was at least 6.4%. Genotyping of only paternal mutation was possible in one of the other two families. This is the first NIPT protocol potentially applicable to any AR monogenic disease with various genotypes, including point mutations.

The main benefit of prenatal diagnosis is the timely management of diseases before or after birth. To date, the two most commonly used methods for prenatal diagnosis have been chorionic villus sampling and amniocentesis, which carries a 1% risk of miscarriage^1^,^2^,^3^. Unless the benefit from diagnosing a certain disease outweighs the risk, it is difficult to justify using these two methods to perform prenatal diagnosis.

Recently, prenatal diagnosis using cell-free fetal DNA (cffDNA) has been developed^4^,^5^,^6^. This method, unlike the two aforementioned methods, provides genetic information of fetuses in a noninvasive manner, as cffDNA can be obtained from the maternal peripheral blood. Hence, this method is regarded as a noninvasive prenatal testing (NIPT). Several studies have demonstrated that fetal aneuploidies and chromosome abnormalities can be detected by NIPT using cffDNA^7^,^8^,^9^. Consequently, monogenic diseases, which may not be fatal but certainly beneficial to diagnose, can be detected. However, NIPT using cffDNA is a technically challenging procedure, because the fetal DNA is indistinguishable from the maternal genomic DNA (gDNA) in the maternal plasma^10^. This challenge can be overcome by measuring the genetic imbalance in the maternal plasma caused by cffDNA.

The genetic imbalance can be detected in two ways: Reconstruction and prediction of the fetal haplotype and direct genotyping of the residue of interest. The former technique is based on massive parallel sequencing (MPS)^11^. The latter is based on digital polymerase chain reaction (dPCR)^4^,^6^. NIPT using targeted MPS technology requires numerous informative single nucleotide polymorphisms (SNPs) around the residue of interest for reconstruction of the fetal haplotypes. This may not be possible in some cases and sometimes recombination of alleles of the fetus should be considered. The second technique mentioned may comparatively be simpler and more straightforward than the first with respect to direct genotyping of the residue of interest. The genetic imbalance in the maternal plasma is measured by dPCR. However, the previous chip-based dPCR does not have sufficient resolution to diagnose general monogenic diseases. Statistical correction, like the Poisson distribution, is required to measure the genetic imbalance^4^,^5^,^6^, reducing the accuracy of NIPT. Since the resolution of dPCR depends on the number and volume of partitions, a greater number of partitions with smaller volume can guarantee higher resolution^12^. Moreover, the previously suggested protocols that employ dPCR to diagnose autosomal recessive (AR) monogenic diseases cannot completely cover AR monogenic diseases with a compound heterozygous genotype^4^,^5^.

For the first time, we utilized picodroplet dPCR to perform NIPT, which generated millions of picoliter-sized droplets. Since there are no droplets with multiple copies of the target in picodroplet dPCR^13^,^14^, statistical compensation is not necessary to measure the genetic imbalance. It leads to a diagnosis with greater accuracy. To the best of our knowledge, no study to date has presented successful NIPT results from using dPCR to diagnose AR monogenic diseases with a compound heterozygous genotype. Although it is technically more challenging, a protocol of NIPT for these diseases is necessary.

Prelingual sensorineural hearing loss (SNHL) usually occurs in an AR fashion. Diverse combinations of genotypes have been documented for prelingual, hereditary SNHL. SNHL requires a timely auditory rehabilitation for the proper development of language skills. Any delay in auditory rehabilitation will hinder language development in subjects with hearing loss^15^. Given this, prelingual SNHL is a good target disease to test a novel comprehensive NIPT approach for various AR genotype combinations. For this purpose, we recruited four families segregating genetically diagnosed AR type prelingual SNHL and expecting a new baby. We developed a protocol of NIPT employing picodroplet dPCR, with an expectation for it to be applicable to diagnose AR diseases with any combination of genotypes.

## Methods

### Subjects and Ethical Considerations

The institutional review boards of both Seoul National University Hospital (IRBY-H-0905–041–281) and Seoul National University Bundang Hospital (IRB-B-1007-105-402 and IRB-B-1508-312-304) approved all procedures used in this study. All subjects provided written informed consent. All methods were performed in accordance with the relevant guidelines and regulations. Four families with the first baby already confirmed to have SNHL due to AR mutations of known deafness genes and an unborn baby (fetus) were included in this study. The causative mutations of SNHL from these four families have previously been documented (the first family (SH123): *SLC26A4* c.1529 T > A (p.V510D)/c.2168 A > G (p.H723R), the second family (SB191): *GJB2* c.299_300delAT (p.H100RfsX14)/c.123 G > A (p.G45E), the third family (SB170): *GJB2* c.235delC homozygote, the fourth family (SB251): *GJB2* c.508_511dupAACG (p.A171EfsX40)/c.257 C > G (p.T86R) (Supplementary Figure S1). NIPT was performed for genotyping of the causative deafness gene from the unborn baby of each family.

### Plasma DNA extraction protocol

Blood samples were collected from all pregnant mothers. At the time of this procedure, the maternal gestational age of the first, second, third, and fourth families was 15, 20, 18, and 10 weeks, respectively. The maternal body weight was 60.0, 58.2, 68.5 and 55.2 kg, respectively. Plasma was centrifuged for 10 min at 2000 g using MACHEREY-NAGEL, NucleoSpin Plasma XS (Germany) kit. We strictly followed the manufacturer’s guidelines using the manual, which involved the extraction of circulating DNA in 1–2 days or freezing of plasma at −20 °C. We used the ‘high sensitivity protocol’, with the exception of the first step. Based on our repeated trials that resulted in severe loss of yield, we added 1.5 ml plasma—not 240 μl plasma, as instructed—to a microcentrifuge tube. The buffer volume was adjusted accordingly.

### gDNA preparation

The gDNA previously obtained from the father, mother, and first baby in each family was fragmented to mimic the plasma DNA using Covaris S220 (Covaris, MA, USA). This fragmented gDNA was used as a constituent of positive and negative control DNAs so that the control DNAs were close to pDNA at least in terms of size. The fragment size of 150 base pair length was confirmed by Bioanalyzer High Sensitivity DNA Chips (Agilent Technologies, CA, USA). DNA concentration was determined using a fluorescence assay of Picogreen (Invitrogen, Grand Island, NY, USA).

### Picodroplet digital PCR (dPCR) methods

RainDrop Digital PCR System (RainDance Technologies Inc., Billerica, MA, USA) was used to assess picodroplet dPCR. In a pre–polymerase chain reaction environment, PCR reaction mixes were combined with primers and probes (the sequences and concentrations of primers and probes are given in Supplementary Table S1) along with 1.25 μl Drop Stabilizer (RainDance Technologies), 12.5 μl TaqMan Genotyping Master Mix (Life Technologies), DNase/RNase-free sterile water, and template DNA (either the minimum 2 ng of plasma DNA or 30 ng of the fragmented gDNA), which made up a total reaction volume of 25 μl. All probes were validated (Supplementary Figure S2). To emulsify the PCR reaction mix, it was loaded onto the RainDrop Source instrument (RainDance Technologies), carefully following the guidelines. Each 25 μl PCR mix was emulsified into 5 pl droplet volumes, partitioning a single molecule of DNA into approximately 5 million droplets. After emulsion, the PCR mixes were placed in a C1000 with deep-well (Bio-Rad) to be amplified, following the protocol outlined in Supplementary Table S2. The thermal cycled samples were loaded onto the RainDrop Sense instrument (RainDance Technologies), identifying the fluorescent intensity of each droplet for two fluorophores (FAM and VIC) simultaneously using a 488 nm laser. After evaluating all the samples, data from the cluster plots were spectrally-compensated and analyzed using the RainDrop Analyst data analysis software, in accordance with the standard procedures. The sample containing the highest mutant titration was used as the control sample to define the gates around the cluster of droplet events. These gates were applied across all evaluated samples within each assay. The same mutant gate was set within all wildtype-only samples, in which the droplets with mutant signals (droplet events that are counted within the mutant gate) were considered false-positive. These false-positive events were subtracted from the total mutant signal when counting the true-positive-mutant events across the samples.

### Noninvasive prenatal testing (NIPT) protocol

The known causative mutations were confirmed by Sanger sequencing from gDNAs of the father, mother, and first baby in each family. Then, NIPT was performed by two-track approach, depending on the homozygosity of the causative deafness mutations in each family.

#### In case of compound heterozygosity for the causative mutation

##### Preparatory step (Validation of our methodology)

The precision and applicability of our protocol were tested prior to genotyping of the fetal DNA. We attempted to evaluate whether consistent values were obtained throughout the repeated measurements of three different samples that were supposed to have mutant and wildtype residues at a ratio of 1:1.1368 (first family) or 1:1 (second family) (Supplementary Table S3). The result was expressed as the mean fraction of mutant sequence over the total reads at the mutated residue with standard deviations (SD). The values obtained by two measurements from the three samples were 0.4667 ± 0.0003, 0.4957 ± 0.0068, and 0.4794 ± 0.0008. SD below 0.0068 and detection of slightly low fraction from the first family ensured that our system can be applicable to genotyping of the fetal DNA (Supplementary Table S3).

Phase I (Genotyping of a paternal mutation): A fraction of the fetal DNA of unborn baby in the maternal plasma DNA (mpDNA) was calculated using either a paternal mutation of our interest or other known SNP exclusively from the paternal gDNA. The paternal mutation or the previously chosen SNP exclusively from paternal gDNA would not exist theoretically in mpDNA unless it had been inherited to the fetus. Firstly, the fraction of signals from the mutant cluster over the total signals from both the wildtype and mutant clusters in mpDNA was measured at the paternal mutant residue by picodroplet dPCR. If the mutant signals from the paternal causative mutant residue was close to nil, then we designed the primers and probes for detection of the paternal gDNA-specific homozygous SNP and calculated the fetal DNA fraction using signals from this SNP. Based on the calculated fraction, we made an artificial DNA mixture simulating the composition of positive and negative controls for the paternal causative mutation: The positive and negative controls account for maternal gDNA artificially containing the gDNA components with and without paternal mutation in the calculated fetal DNA fraction, respectively. The positive control was a mixture of gDNAs from the mother and first baby, represented as a ratio of the fetal DNA to the total mpDNA; the negative control comprised of the plasma DNA from any subjects without paternal mutation. Next, a fraction of signals from the mutant cluster over the total signals from both the wildtype and mutant clusters in positive and negative controls was measured at the paternal mutant residues, using picodroplet dPCR.

We determined whether the fetal DNA carried a paternal mutation by analyzing the direction of genetic imbalance caused by cffDNA between the wildtype and mutant alleles at the paternal mutant residues in mpDNA—either toward the positive controls or negative controls. This was quantified by calculating and comparing the sum of the log-likelihood of the study samples under the assumption that they followed a normal distribution of the positive and negative controls, respectively. The fetus was diagnosed as having a paternal mutation if the sum of the log-likelihood of the study samples following a normal distribution of the positive control was greater than that of the negative control. Otherwise, the fetus was diagnosed as containing no paternal mutation. A calculation of the test statistic was not necessary as the acquired data were well discriminated enough to make the uncertainty of likelihood negligible.

Phase II (Genotyping of a maternal mutation): Next, we checked for whether the fetal DNA had a maternal mutation. Theoretically, the ratio between the signals from the wildtype and mutant clusters at the maternal mutant residues is expected to be 1:1 without the fetal DNA. Given that the fetal DNA is contained in mpDNA, we aimed to detect any deviation from the expected ratio of 1:1. Both positive and negative controls for maternal mutation were also generated as calculated above, considering the fraction of fetal DNA in mpDNA. The positive control was a mixture of maternal gDNA harboring the mutation in a heterozygous state and first baby’s gDNA as a ratio of the fetal DNA to the total plasma DNA. The negative control included maternal gDNA mixed with gDNA not harboring the maternal mutation as a ratio of the fetal DNA proportion. Next, a fraction of signals from the mutant cluster over the total signals from both the wildtype and mutant clusters in mpDNA with unknown fetal DNA genotypes, positive controls, and negative controls was measured at the maternal mutant residues, using picodroplet dPCR.

**Figure 1:**
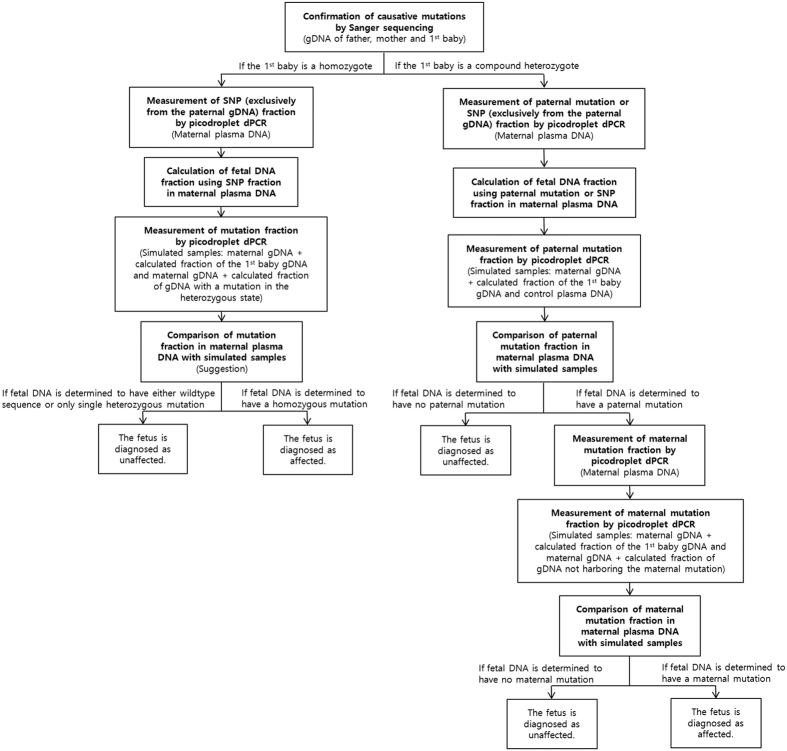
Protocol of noninvasive prenatal diagnosis.

The same discriminant analysis was done as in the first phase. The fetus was diagnosed as containing a maternal mutation if the sum of the log-likelihood of the study samples following a normal distribution of the positive control was greater than that of the negative control. Otherwise, the fetus was diagnosed as containing no maternal mutation (Fig. 1).

### In case of homozygosity for the causative mutation

If the first baby carried a homozygous mutation, genotyping was tried in a single stage. In this situation, a fraction of the fetal DNA in mpDNA was calculated from the fraction of a previously chosen SNP, which was documented to exist exclusively from the paternal gDNA. Based on this result, positive and negative control samples for the homozygous mutation were generated with consideration to the calculated fraction of the fetal DNA. The positive control was a mixture between maternal gDNA with a mutation in the heterozygous state and the first baby’s gDNA harboring a mutation in the homozygous state as a ratio of the fetal DNA to the total plasma DNA. The negative control was a mixture of maternal gDNA and gDNA with a mutation in the heterozygous state as a ratio of the fetal DNA proportion. A fraction of signals from the mutant cluster over the total signals from both the wildtype and mutant clusters in mpDNA with unknown fetal DNA genotypes, positive controls, and negative controls was measured, using picodroplet dPCR.

The same discriminant analysis was performed as above. The fetus was diagnosed with having a homozygous mutation if the sum of the log-likelihood of the study samples following a normal distribution of the positive controls was greater than that of the negative controls. Otherwise, the fetus was diagnosed as containing no mutation or carrying a mutation in the heterozygous state. Clinically, the latter was expected to be unaffected (Fig. 1).

### Genetic study for confirmation of fetal genotype

Sanger sequencing of gDNA from buccal mucosa of the second baby for targeted gene after birth served as a gold standard for the genotyping and the predicted fetal genotypes were checked against these Sanger sequencing results.

## Results

### Prediction of fetal genotypes by our NIPT protocol

**Figure 2:**
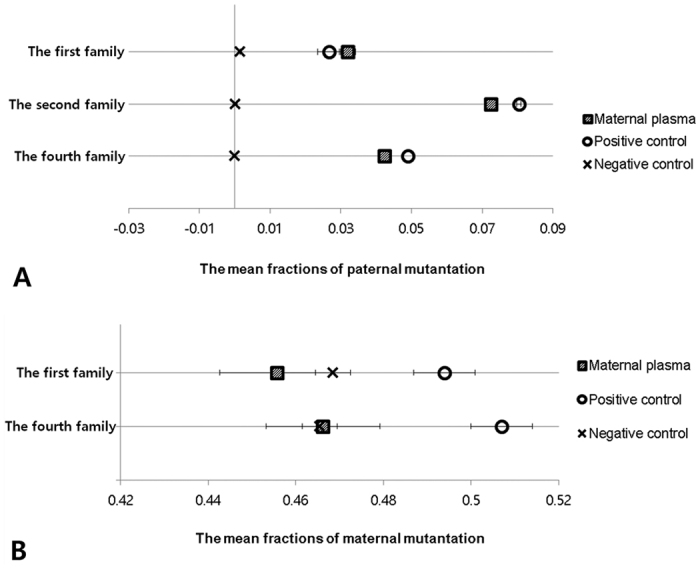
The mean fractions of paternal and maternal mutations. (**A**) The mean fractions of a paternal mutation in the maternal plasma DNA, and the positive and negative controls of the first family were 0.0319, 0.0267 and 0.0015, respectively. The mean fractions of a paternal mutation in maternal plasma DNA, and the positive and negative controls of the second family were 0.0725, 0.0804 and 0.0002, respectively. The mean fractions of a paternal mutation in maternal plasma DNA, and the positive and negative controls of the fourth family were 0.0423, 0.0490 and 0.0000, respectively. (**B**) The mean fractions of a maternal mutation in maternal plasma DNA, and the positive and negative controls of the first family were 0.4557, 0.4939 and 0.4685, respectively. The mean fractions of a maternal mutation in maternal plasma DNA, and the positive and negative controls of the fourth family were 0.4662, 0.5070 and 0.4655, respectively.

**Figure 3:**
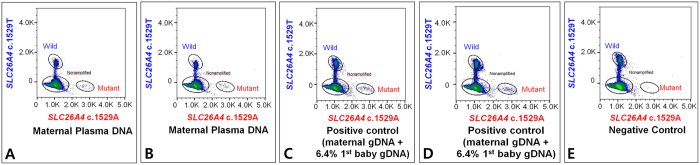
Two-dimensional histogram of the paternal mutation (*SLC26A4* c.1529 T > A (p.V510D)) in maternal plasma DNA (**A,B**), and positive (**C,D**) and negative (**E**) controls of the first family.

In the first family, the father was a carrier of *SLC26A4* c.1529 T > A (p.V510D), mother was a carrier of *SLC26A4* c.2168 A > G (p.H723R), and the first baby was a compound heterozygote of *SLC26A4* c.1529 T > A (p.V510D)/c.2168 A > G (p.H723R). The mean fraction of paternal mutation in mpDNA was 0.0319. A fraction of the fetal DNA in mpDNA was 6.4% ((64 × 2)/(1845 + 64) + (60 × 2)/(1921 + 60))/2). The mean fractions of paternal mutation in positive and negative controls were 0.0267 and 0.0015, respectively (Table 1 and Figs 2A and 3). Sequentially, the fetus was diagnosed as having a paternal mutation by discriminant analysis (sum of the log-likelihood: positive control, 6.8677; negative control, -infinity) (Table 2). The mean fractions of maternal mutation in mpDNA, as well as positive and negative controls were calculated as 0.4557, 0.4939, and 0.4685, respectively (Table 1, Fig. 2B and Supplementary Figure S3). Sequentially, the fetus was diagnosed as having no maternal mutation by discriminant analysis (sum of the log-likelihood: positive control, −36.5065; negative control, −12.2726) (Table 2). The fetus was diagnosed as unaffected.

In the second family, the father was a carrier of *GJB2* c.299_300delAT (p.H100Rfs*14), mother was a carrier of *GJB2* c.123 G > A (p.G45E), and the first baby was a compound heterozygote of *GJB2* c.299_300delAT (p.H100Rfs*14)/c.123 G > A (p.G45E). The mean fraction of paternal mutation in mpDNA was 0.0725. The fraction of fetal DNA was 14.5% ((93 * 2)/(1190 + 93)). The mean fraction of paternal mutation in positive and negative controls were 0.0804, and 0.0002, respectively (Table 1, Fig. 2A and Supplementary Figure S4). Sequentially, the fetus was diagnosed as having a paternal mutation by discriminant analysis (sum of the log-likelihood: positive control, −89.6183; negative control, -infinity) (Table 2). The mean fractions of maternal mutation in mpDNA, as well as positive and negative controls were calculated as 0.3957, 0.4710, and 0.3904, respectively (Table 1 and Supplementary Figure S5). However, from the result of picodroplet dPCR using the probe for maternal mutation, mpDNA did not show a distinct cluster for the wildtype. The tail around the cluster resulted in an ambiguous wildtype count (Supplementary Figure S5A and B). Although the fraction of signals from the maternal mutant cluster over the total signals from both the wildtype and mutant clusters in mpDNA were achieved through a data analysis software, we were unable to determine whether the fetus had a maternal mutation (Table 1 and Supplementary Figure S5). Therefore, only a partial prenatal diagnosis of the fetus was made.

In the third family, both the father and mother were carriers of *GJB2* c.235delC, and the first baby was a *GJB2* c.235delC homozygote. The mean fraction of SNP, detected exclusively from the paternal gDNA, in mpDNA was 0.014 (Table 1 and Supplementary Figure S6). The fraction of the fetal DNA in mpDNA was only 2.7% (((24 * 2)/(2507 + 24) + (10 * 2)/(560 + 10))/2). The genotype status of the fetus could not be predicted due to a low fraction of cffDNA.

In the fourth family, the father was a carrier of *GJB2* c.508_511dupAACG (p.A171Efs*40), the mother was a carrier of *GJB2* c.257 C > G (p.T86R), and the first baby was a compound heterozygote of *GJB2* c.508_511dupAACG (p.A171Efs*40)/c.257 C > G (p.T86R). The mean fraction of paternal mutation in mpDNA was 0.0423. The fraction of the fetal DNA in mpDNA was 8.5% ((41 × 2)/(899 + 41) + (35 × 2)/(820 + 35))/2). The mean fraction of the paternal mutation in positive and negative controls was 0.0490 and 0.0000, respectively (Table 1, Fig. 2A and Supplementary Figure S8). Sequentially, the fetus was diagnosed as having a paternal mutation by discriminant analysis (sum of the log-likelihood: positive control, −16.3308; negative control, −1050.1540) (Table 2). The mean fraction of maternal mutation in mpDNA, as well as positive and negative controls were calculated as 0.4662, 0.5070, and 0.4655, respectively (Table 1, Fig. 2B and Supplementary Figure S9). Sequentially, the fetus was diagnosed as having no maternal mutation by discriminant analysis (sum of the log-likelihood: positive control, -infinity; negative control, 10.4021) (Table 2). The fetus was diagnosed as unaffected.

### Confirmation of fetal genotype by Sanger sequencing

**Figure 4:**
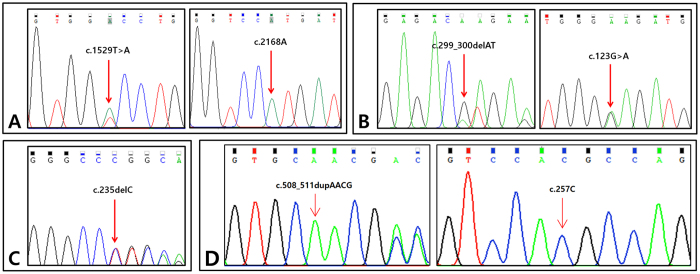
Genetic study for confirmation of fetal genotype. (**A**) Sanger sequencing traces of the second-born baby of the first family: *SLC26A4* c.1529 T > A (p.V510D) single heterozygote. (**B**) Sanger sequencing traces of the second-born baby of the second family: *GJB2* c.299_300delAT (p.H100Rfs*14)/c.123 G > A (p.G45E). (**C**) Sanger sequencing traces of the second-born baby of the third family: *GJB2* c.235delC single heterozygote. (**D**) Sanger sequencing traces of the second-born baby of the fourth family: *GJB2* c.508_511dupAACG (p.A171Efs*40) single heterozygote.

Sanger sequencing from the second baby in the first, second, third, and fourth families confirmed the following: a single heterozygote of *SLC26A4* c.1529 T > A (p.V510D), compound heterozygote of *GJB2* c.299_300delAT (p.H100Rfs*14) and c.123 G > A (p.G45E), a single heterozygote of *GJB2* c.235delC, and a single heterozygote *GJB2* c.508_511dupAACG (p.A171Efs*40) (Fig. 4A–D). The prenatal diagnosis of paternal and maternal mutations for the second baby in the first and fourth family and paternal mutation for the second baby in the second family was correct (Table 3).

## Discussion

Up until recently, prenatal diagnosis, despite having many advantages for certain diseases, has never been performed on a regular basis. This was the case because prior methods—chorionic villus sampling and amniocentesis—were highly invasive with associated risks. However, with the discovery of cffDNA in the peripheral blood of pregnant women, which provides genetic information of the fetus, prenatal testing became more feasible^16^.

NIPT using cffDNA started from determination of fetal sex^16^ and RhD status^17^,^18^. The indication of NIPT was extended to aneuploidies, which is relatively an easier target. In 2007, it was detected that genetic material derived from chromosome 21 increased in the plasma of pregnant woman carrying a fetus with trisomy 21^19^,^20^. Prenatal diagnosis of trisomy 21 was performed through measuring the genomic representation of chromosome 21 with random MPS^21^,^22^. With the improvement in technology and strategy, the indication criteria for NIPT have expanded. Monogenic diseases have been diagnosed using NIPT^4^,^5^,^23^,^24^.

Our method using picodroplet dPCR, compared with previous methods of NIPT—MPS and chip-based dPCR, has several merits in diagnosing monogenic diseases. First, the biggest difference is that MPS deducts the fetal haplotypes through sequencing numerous SNPs around the residue of interest^25^,^26^,^27^,^28^,^29^. Conversely, our method sequences a residue of interest directly even if a mutation exists in a homozygous fashion. Sequentially, the method using MPS requires a lot of time and labor, and it poses concerns about chromosomal recombination inevitably, while our method offers greater clarity and simplicity. Additionally, if an efficient probe for a certain founder allele has been constructed previously, early diagnosis can be facilitated in many cases. Second, to date, several researchers have attempted NIPT employing chip-based dPCR for the following diseases: beta-thalasemia, hemophilia, and sickle cell anemia. Lun *et al*. detected fetal alleles of the beta-thalasemia mutation that were inherited from the mother^30^. Tsui *et al*. successfully reported the applicability of NIPT in detecting a female carrier of hemophilia with male fetuses^6^. Moreover, Barrett *et al*. successfully reported the applicability of NIPT for sickle cell anemia^4^. Although these studies showed successful results, they had limitations. As chip-based dPCR has less than 800 partitions, there must be a chamber containing multiple copies of a target. Containing one or no target copy in one partition is the major premise of dPCR to achieve high resolution. Although the Poisson distribution has been used to overcome this weakness, it can only compensate to a limited extent, reducing the accuracy of NIPT. To overcome this, we employed picodroplet dPCR instead of chip-based dPCR. To the best of our knowledge, this is the first study to use picodroplet dPCR in NIPT. Picodroplet dPCR generates millions of picoliter droplets. One droplet contains one or no target copy, and there is no droplet containing multiple copies of a target^13^,^14^. Consequently, statistical compensation, such as the Poisson distribution, is not needed to get the actual number of target molecules. This contributes to a more accurate prenatal diagnosis.

Additionally, we utilized positive and negative control samples. The results of the tested samples were compared with that of the control samples; prenatal diagnosis was made based on a discriminant analysis. In previous studies, however, they calculated the theoretically expected proportion of the mutant and wildtype alleles, comparing the value obtained from the tested sample against the calculated value^4^,^6^. The calculated value cannot reflect the effect of intrinsic variables of the experiment. There must be a difference between the calculated value and the value from a real sample, like the control sample in our study. Considering that dPCR requires a highly sensitive technique on a molecular-level, simulation with control samples could contribute to greater accuracy. As such, an accurate prenatal diagnosis can be made for point mutations in a compound heterozygous fashion using our novel protocol.

It is, however, worth noting that in our second and third families, a complete prenatal diagnosis was not achieved. The failure of NIPT in the third family was attributed to low cffDNA fraction (2.7%). A previous study using dPCR also reported that a higher cffDNA fraction was required to make a correct prenatal diagnosis^30^. Barrett *et al*. reported that 100% accuracy of NIPT was achieved at a cffDNA fraction of greater than 7%^4^. The effect of cffDNA fraction showed a similar tendency in NIPT using MPS. Yoo *et al*. reported that the lowest cffDNA fraction allowing successful NIPT with MPS was 5.8%^29^. Although New *et al*. reported a successful prenatal diagnosis with cffDNA fraction of 1.4%^28^, it was for a paternal mutation. In our results, the presence of SNP exclusively for a paternal DNA was detected in mpDNA under the cffDNA fraction of 2.7% (third family), which suggests that prenatal prediction of a paternal mutation is much more feasible compared with a maternal mutation even under a low fraction of cffDNA among mpDNA. NIPT using cffDNA is based on the measurement of imbalance in mpDNA caused by cffDNA. Consequently, if the fraction of cffDNA is too small to make a detectable imbalance in mpDNA, a prenatal diagnosis would not be possible. High enough cffDNA fraction seems to be one of the most important prerequisites for NIPT that uses dPCR or MPS. A cffDNA fraction is suggested to be influenced by gestational age and maternal body weight^31^,^32^. As cffDNA fraction tends to rise with increasing gestational age, this should be considered when performing NIPT at early gestational ages. In addition, as mother’s weight increases, maternal blood volume increases, which lowers the cffDNA fraction. In this present study, the mother of the third family, who was unable to successfully complete NIPT, was heavier that the other three mothers. As speculated, higher maternal body weight may indeed negatively influence the accuracy of NIPT.

Although we modified the probes for maternal mutation several times in the second family, the tail around the cluster of the wildtype was not removed (Supplementary Figure S5A and B), and a prenatal diagnosis for maternal mutation was not achieved. This might be attributed to the characteristics of the individual plasma DNA or a peculiar reaction between the individual plasma DNA and the probe. However, a successful prenatal diagnosis was achieved for a paternal mutation. In this case, NIPT using MPS could be an alternative. As NIPT utilizing dPCR or MPS has its own advantages and disadvantages, they can be complementary to one another. Therefore, an appropriate use of dPCR or MPS will contribute to a more accurate prenatal diagnosis.

In summary, we were able to make a successful prediction of the fetal genotype for AR monogenic diseases that result from point mutations by utilizing picodroplet dPCR in NIPT. This was possible particularly in cases where we were able to identify a distinct cluster for each genotype and where the fractions of cffDNA in mpDNA was at least 6.4%. Moreover, in other cases, at least partial genotyping was possible.

For the first time in the literature, we report a successfully developed protocol of NIPT for the genotyping of compound heterozygous point mutations of AR monogenic diseases by coupling the dPCR technique with a sophisticated statistical analysis. This protocol is applicable to any AR monogenic diseases with various genotypes, including point mutations; it is not limited for a specific disease if the fraction of cffDNA is higher than a certain level. Improved techniques in obtaining distinctive clusters for each genotype would make this protocol popular. With the incorporation of our novel protocol, NIPT can become a popular tool for prenatal diagnosis, making it possible to prenatally diagnose a number of AR monogenic diseases with various genotypes. Through this prenatal diagnosis, timely management of diseases leading to better lives is feasible. This would pave the way for the establishment of a widely used prenatal diagnosis method in the near future.

## Additional Information

**How to cite this article**: Chang, M. Y. *et al*. Development of novel noninvasive prenatal testing protocol for whole autosomal recessive disease using picodroplet digital PCR. *Sci. Rep.* **6**, 37153; doi: 10.1038/srep37153 (2016).

**Publisher's note:** Springer Nature remains neutral with regard to jurisdictional claims in published maps and institutional affiliations.

## Supplementary Material

Supplementary Information

## Figures and Tables

**Table 1:** The results of noninvasive prenatal testing using picodroplet digital PCR.

Family	Probe	Sample	Intact drops	Wild type	Mutant (SNP)	Corresponding histogram	Fraction of a mutant sequence over the wildtype+mutant sequence at the mutant residue(SNP)	Mean fraction of a mutant sequence over the wildtype+mutant sequence at the mutant residue(SNP) (±SD)
The first family	Paternal mutation, *SLC26A4* c.1529 T > A (p.V510D)	Maternal plasma DNA	4679781	1845	64	Figure 3(A)	0.0335	0.0319 ± 0.0023
			4315445	1921	60	Figure 3(B)	0.0303	
		Positive control (maternal gDNA + 6.4% 1st baby gDNA)	4715315	5421	135	Figure 3(C)	0.0243	0.0267 ± 0.0033
			4746722	5988	179	Figure 3(D)	0.0290	
		Negative control (plasma DNA from the subject who has no paternal mutation)	3911735	4515	7	Figure 3(E)	0.0015	0.0015
	Maternal mutation, *SLC26A4* c.2168 A > G (p.H723R)	Maternal plasma DNA	4515957	973	854	Supplementary Figure S3(A)	0.4674	0.4557 ± 0.0130
			4236100	1076	909	Supplementary Figure S3(B)	0.4579	
			5012319	1464	1158	Supplementary Figure S3(C)	0.4416	
		Positive control (maternal gDNA + 6.4% 1st baby gDNA)	3961002	1480	1481	Supplementary Figure S3(D)	0.5002	0.4939 ± 0.0070
			3749613	1413	1411	Supplementary Figure S3(E)	0.4996	
			4234397	1661	1582	Supplementary Figure S3(F)	0.4878	
			4296400	1648	1570	Supplementary Figure S3(G)	0.4879	
		Negative control (maternal gDNA + 6.4% paternal gDNA)	4212043	1814	1561	Supplementary Figure S3(H)	0.4625	0.4685 ± 0.0040
			4249353	1735	1545	Supplementary Figure S3(I)	0.4710	
			4161255	1687	1495	Supplementary Figure S3(J)	0.4698	
			3703065	1514	1345	Supplementary Figure S3(K)	0.4704	
The second family	Paternal mutation, *GJB2* c.299_300delAT (p.H100Rfs*14)	Maternal plasma DNA	3789570	1190	93	Supplementary Figure S4(A)	0.0725	0.0725
		Positive control (maternal gDNA + 14.5% 1st baby gDNA)	4092063	3503	308	Supplementary Figure S4(B)	0.0808	0.0804 ± 0.0006
			4383191	3599	313	Supplementary Figure S4(C)	0.0800	
		Negative control (plasma DNA from the subject who has no paternal mutation)	4570705	1185	1	Supplementary Figure S4(D)	0.0008	0.0002 ± 0.0004
			4234400	1004	0	Supplementary Figure S4(E)	0.0000	
			4406461	1631	0	Supplementary Figure S4(F)	0.0000	
			4534600	1719	0	Supplementary Figure S4(G)	0.0000	
	Maternal mutation, *GJB2* c.123 G > A (p.G45E)	Maternal plasma DNAPositive control (maternal gDNA + 14.5% 1st baby gDNA)	4265309	835	545	Supplementary Figure S5(A)	0.3949	0.3957 ± 0.0011
			4411174	656	431	Supplementary Figure S5(B)	0.3965	
			4686982	2702	2458	Supplementary Figure S5(C)	0.4764	0.4710 ± 0.0080
			3837244	2037	1862	Supplementary Figure S5(D)	0.4776	
			4100991	2309	2049	Supplementary Figure S5(E)	0.4702	
			4118807	2266	1931	Supplementary Figure S5(F)	0.4601	
		Negative control (maternal gDNA + 14.5% paternal gDNA)	4365336	3050	2012	Supplementary Figure S5(G)	0.3975	0.3904 ± 0.0233
			4636365	3092	2241	Supplementary Figure S5(H)	0.4202	
			3969051	2685	1605	Supplementary Figure S5(I)	0.3741	
			3869329	2778	1630	Supplementary Figure S5(J)	0.3698	
The third family	SNP exclusively for father, *CDH23* c.366 T > C (p.V122V)	Maternal plasma DNA	2978242	2507	24	Supplementary Figure S6(A)	0.0095	0.0135 ± 0.0057
			4199506	560	10	Supplementary Figure S6(B)	0.0175	
	Paternal and maternal mutation, *GJB2* c.235delC	Maternal plasma DNA	1801793	155	107	Supplementary Figure S7(A)	0.4084	0.4794 ± 0.0473
			3003431	594	603	Supplementary Figure S7(B)	0.5038	
			4617054	281	286	Supplementary Figure S7(C)	0.5044	
			4602474	286	287	Supplementary Figure S7(D)	0.5009	
The fourth family	Paternal mutation, *GJB2* c.508_511dupAACG (p.A171Efs*40)	Maternal plasma DNA	4505527	899	41	Supplementary Figure S8(A)	0.0436	0.0423 ± 0.0019
			4483070	820	35	Supplementary Figure S8(B)	0.0409	
		Positive control (maternal gDNA + 8.5% 1st baby gDNA)	4683171	5397	278	Supplementary Figure S8(C)	0.0490	0.0490
		Negative control (plasma DNA from the subject who has no paternal mutation)	4528755	867	0	Supplementary Figure S8(D)	0.0000	0.0000
			4551158	865	0	Supplementary Figure S8(E)	0.0000	
	Maternal mutation, *GJB2* c.257 C > G (p.T86R)	Maternal plasma DNA	4517281	1019	894	Supplementary Figure S9(A)	0.4673	0.4662 ± 0.0015
			4816064	1090	948	Supplementary Figure S9(B)	0.4652	
		Positive control (maternal gDNA + 8.5% 1st baby gDNA)	4733818	4660	4804	Supplementary Figure S9(C)	0.5076	0.5070 ± 0.0008
			4648914	4529	4647	Supplementary Figure S9(D)	0.5064	
		Negative control (maternal gDNA + 8.5% paternal gDNA)	4475033	4588	4010	Supplementary Figure S9(E)	0.4664	0.4655 ± 0.0013
			4730972	4962	4305	Supplementary Figure S9(F)	0.4646	

SNP, single nucleotide polymorphism; SD, standard deviation; gDNA, genomic DNA.

**Table 2:** Calculation of the sum of log-likelihood that the fraction of signal from mpDNA will follow a normal distribution of the positive controls vs. the negative controls.

		Positive control	Negative control
The first family	Paternal mutation	6.8677	-Infinity
	Maternal mutation	−36.5065	−12.2726
The second family	Paternal mutation	−89.6183	-Infinity
The fourth family	Paternal mutation	−16.3308	−1050.1540
	Maternal mutation	-Infinity	10.4021

**Table 3:** Comparison of the results of noninvasive prenatal testing and Sanger sequencing.

Family	Paternal mutation	Maternal mutation	Final diagnosis
	*Prediction/Confirmed*	*Prediction/Confirmed*	*Prediction/Confirmed*
The first family	Mutant/Mutant	Wildtype/Wildtype	Normal/Normal
The second family	Mutant/Mutant	Not possible/Mutant	Not possible/Deaf
The third family	Not possible/Single heterozygote	Not possible/Single heterozygote	Not possible/Normal
The fourth family	Mutant/Mutant	Wildtype/Wildtype	Normal/Normal
