# Supplementary material for: Development of novel noninvasive prenatal testing protocol for whole autosomal recessive disease using picodroplet digital PCR
Source: Sci Rep. 2016 Dec 7;6:37153. doi: 10.1038/srep37153 (PMC5141438; doi:10.1038/srep37153)
Supplement: Supplementary Information [file srep37153-s1.pdf]

# Supplementary Information

**Development of novel noninvasive prenatal testing protocol for whole autosomal recessive disease using picodroplet digital PCR**

Mun Young Chang[1], Ah Reum Kim[2], Min Young Kim[3], Soyoung Kim[4], Jinsun Yoon[5], Jae Joon Han[3], Soyeon Ahn[6], Changsoo Kang[7] & Byung Yoon Choi[3,8,*]

[1] Department of Otorhinolaryngology-Head and Neck Surgery, Chung-Ang University College of Medicine, 102 Heukseok-ro, Dongjak-gu, Seoul, 06973, Republic of Korea.

[2] Department of Otorhinolaryngology, Seoul National University Hospital, Seoul national University College of Medicine, 101 Daehak-ro, Jongno-gu, Seoul 03080, Republic of Korea.

[3] Department of Otorhinolaryngology, Seoul National University Bundang Hospital, 82 Gumi-ro 173 beon-gil, Bundang-gu, Seongnam, 13620, Republic of Korea.

[4] LAS Inc., 16 Arayuk-ro, Gimpo, 10136, Republic of Korea.

[5] Bio-Medical Science Co., Ltd., BMS Bldg., 22 Yeoksam-ro 7-gil, Gangnam-gu, Seoul 06244, Republic of Korea.

[6] Medical Research Collaborating Center, Seoul National University Bundang Hospital, 82 Gumi-ro 173 beon-gil, Bundang-gu, Seongnam, 13620, Republic of Korea.

[7] Department of Biology and Research Institute of Basic Sciences, College of Natural Sciences, Sungshin Women's University, Seoul, 01133, Republic of Korea.

[8] Wide River Institute of Immunology, Seoul National University College of Medicine, 101 Dabyeonbat-gil, Hwachon-myeon, Hongcheon, 25159, Republic of Korea.

* Correspondence to : Byung Yoon, Choi, MD, PhD
Department of Otorhinolaryngology, Seoul National University Bundang Hospital, 82 Gumi-ro 173 beon-gil, Bundang-gu, Seongnam 463-707, Korea, Tel: +82-31-787-7406; Fax: +82-31-787-4057;
E-mail: choiby@snubh.org, choiby2010@gmail.com

**Supplementary Table S1.** The sequences and concentrations of primers and probes

| Name | Sequence(5' to 3') | Conc. Used for RainDrop(μM) | Anneal Temp. |
|---|---|---|---|
| c.1529T>A-F | GCTGGCCTTATATTTGGACTGTTGA | 0.9 | 62 |
| c.1529T>A-R | GGAAATCTGGGTTTTACGTTACTCACA | 0.9 | |
| c.1529T>A-WT Probe | VIC-TGAACTCTCAGGACCACAG-NFQ | 0.2 | |
| c.1529T>A-MT Probe | FAM-TGAACTCTCAGGTCCACAG-NFQ | 0.2 | |
| c.2168A>G-F | CGGGTTCTTTGACGACAACATTAG | 0.35 | 58 |
| c.2168A>G-R | CCCTCTTGAGATTTCACTTGGTTCT | 0.35 | |
| c.2168A>G-WT Probe | VIC-TGACGGTCCATGATGCT-NFQ | 0.2 | |
| c.2168A>G-MT Probe | FAM-TGACGGTCTGTGATGCT-NFQ | 0.2 | |
| c.299_300delAT-F | GCTCCTAGTGGCCATGCA | 0.9 | 58 |
| c.299_300delAT -R | TCACTCTTTATCTCCCCCTTGATGA | 0.9 | |
| c.299_300delAT-WT Probe | VIC–CTCTTCTTCTCATGTCTCC-NFQ | 0.2 | |
| c.299_300delAT-MT Probe | FAM–CCTCTTCTTCTCGTCTCC-NFQ | 0.2 | |
| c.123G>A-F | TCGTTGTGGCTGCAAAGGA | 0.9 | 58 |
| c.123G>A-R | CAGGGTGTTGCAGACAAAGTC | 0.9 | |
| c.123G>A-WT Probe | VIC–CCTGCTCATCTCCCCACAC-NFQ | 0.2 | |
| c.123G>A-MT Probe | FAM–CCTGCTCATCTTCCCACAC-NFQ | 0.2 | |
| c.T366C-F | Commercial product | 0.9 | 58 |
| c.T366C-R | (Thermo Fisher Scientific Inc. | 0.9 | |
| c.T366C-WT Probe | Assay ID: C_25592461_10 | 0.2 | |
| c.T366C-MT probe | rs number: rs3802720) | 0.2 | |
| c.235delC-F | CCATCTCCCACATCCGGC | 0.35 | 60 |
| c.235delC-R | CACGTGCATGGCCACTAG | 0.35 | |
| c.235deC-WT Probe | VIC-AGCTGCAGGGCCCAT-NFQ | 0.2 | |
| c.235delC-MT probe | FAM-ATCAGCTGCAGGCCCAT-NFQ | 0.2 | |
| c.508_511dupAACG-F | TCATGTACGACGGCTTCTCC | 0.35 | 62℃ |
| c.508_511dupAACG-R | GGACACAAAGCAGTCCACAG | 0.35 | |
| c.508_511dupAACG-WT probe | VIC_GAAGTGCAACGCCTGGCCTTG_NFQ | 0.2 | |
| c.508_511dupAACG-MT probe | 6-FAM_GAAGTGCAACGAACGCCTG_NFQ | 0.2 | |
| c.257C>G-F | GGCCCTGCAGCTGATCTT | 0.35 | 58℃ |
| c.257C>G-R | CGTGCATGGCCACTAGGA | 0.35 | |
| c.257C>G-WT probe | VIC_CGCTGGCGTGGACA_NFQ | 0.2 | |
| c.257C>G-MT probe | 6-FAM_ CGCTGGCCTGGACA_NFQ | 0.2 | |

Conc, concentration; Temp, temperature.

**Supplementary Table S2.** The protocol of polymerase chain reaction

| Step | Temp | Time | Cycles |
|---|---|---|---|
| Polymerase activation | 95 ℃ | 10 min | 1 |
| Denaturation | 95 ℃ | 15 sec | 45 |
| Annealing & Extension | *Depending on target | 1 min | 45 |
| Incubation | 98 ℃ | 10 min | 1 |
| Final Hold | 12 ℃ | Hold | |

* A slow ramping speed (0.5◦C) was used during cooling from the denaturation step to the annealing step.

Temp, temperature.

**Supplementary Table S3.** The results of picodroplet digital PCR for confirmation of the methodological precision.

| Family | Probe | Sample | Intact drops | Wild type | Mutant | Fraction of a mutant sequence over the wildtype + mutant sequence at the mutant residue | Mean fraction of a mutant sequence over the wildtype + mutant sequence at the mutant residue (±SD) |
|---|---|---|---|---|---|---|---|
| The first family | Maternal mutation, SLC26A4 c.2168A>G (p.H723R) | Simulated sample (maternal gDNA + 6.4% paternal gDNA) | 4209382 | 2458 | 2153 | 0.4669 | 0.4667 ±0.0003 |
| | | | 3513864 | 2146 | 1876 | 0.4664 | |
| The second family | Paternal mutation, *GJB2* c.299_300delAT (p.H100Rfs*14) | Paternal gDNA | 4105976 | 2147 | 2151 | 0.5000 | 0.4957 ±0.0068 |
| | | | 4350903 | 2431 | 2344 | 0.4909 | |
| | Maternal mutation, *GJB2* c.123G>A (p.G45E) | Maternal gDNA | 4566718 | 2595 | 2395 | 0.4800 | 0.4794 ±0.0008 |
| | | | 4248610 | 2418 | 2222 | 0.4789 | |

SD, standard deviation; gDNA, genomic DNA.

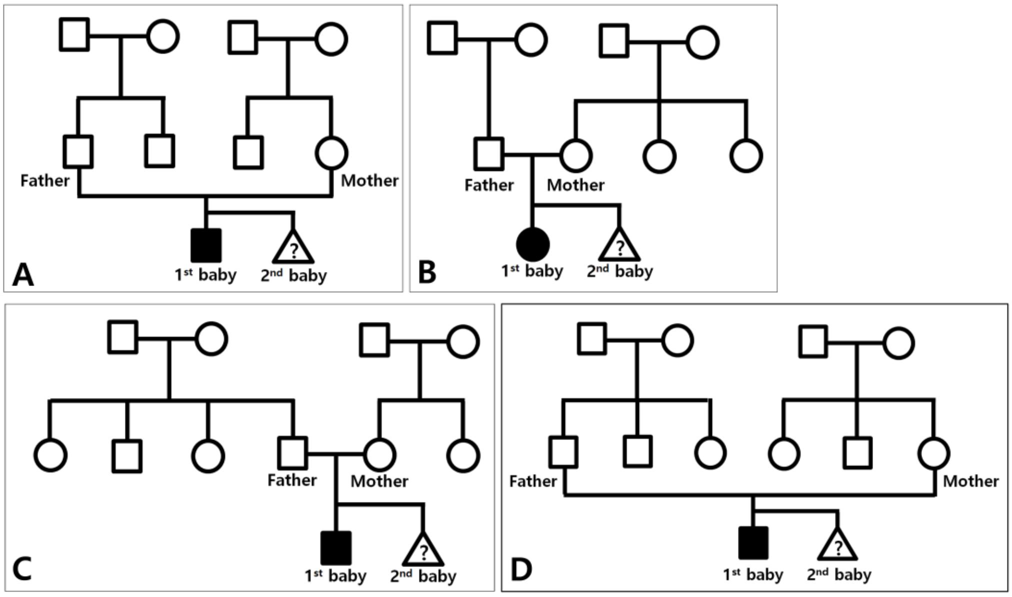


**Supplementary Figure S1.** Pedigree of four families. (A) Sanger sequencing traces of the first family. Father: *SLC26A4* c.1529T>A (p.V510D) carrier, Mother: *SLC26A4* c.2168A>G (p.H723R) carrier, the first baby: *SLC26A4* c.1529T>A (p.V510D) / c.2168A>G (p.H723R). (B) Sanger sequencing traces of the second family. Father: *GJB2* c.299_300delAT (p.H100Rfs*14) carrier, Mother: *GJB2* c.123G>A (p.G45E) carrier, the first baby: *GJB2* c.299_300delAT (p.H100Rfs*14) / c.123G>A (p.G45E). (C) Sanger sequencing traces of the third family. Father and mother: *GJB2* c.235delC carriers, the first baby: *GJB2* c.235delC homozygote. (D) Sanger sequencing traces of the fourth family. Father: *GJB2* c.508_511dupAACG (p.A171Efs*40) carrier, Mother: *GJB2* c.257C>G (p.T86R) carrier, the first baby: *GJB2* c.508_511dupAACG (p.A171Efs*40) / c.257C>G (p.T86R).

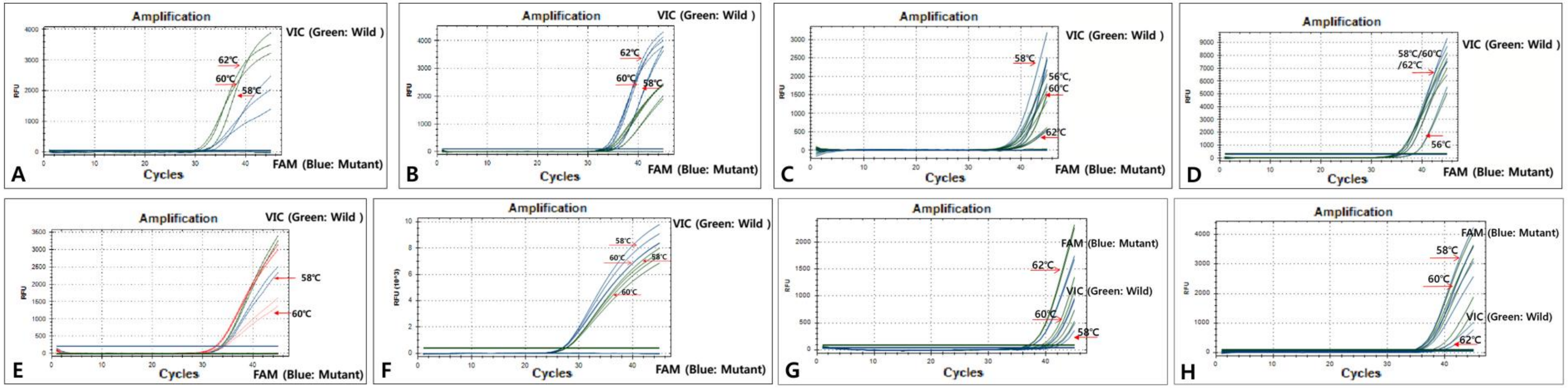


**Supplementary Figure S2.** Validation of probes. The target was detected after less than 40 cycles. (A) The probe for paternal mutation in the first family (*SLC26A4* c.1529T>A (p.V510D)), (B) The probe for maternal mutation in the first family (*SLC26A4* c.2168A>G (p.H723R)), (C) The probe for paternal mutation in the second family (*GJB2* c.299_300delAT (p.H100Rfs*14)), (D) The probe for maternal mutation in the second family (*GJB2* c.123G>A (p.G45E)), (E) The probe for SNP exclusively for the father in the third family (*CDH* c.366C>T (p.V122V)), (F) The probe for paternal and maternal mutation in the third family (*GJB2* c.235delC), (G) The probe for paternal mutation in the fourth family (*GJB2* c.508_511dupAACG (p.A171Efs*40)), (H) The probe for maternal mutation in the fourth family (*GJB2* c.257C>G (p.T86R))

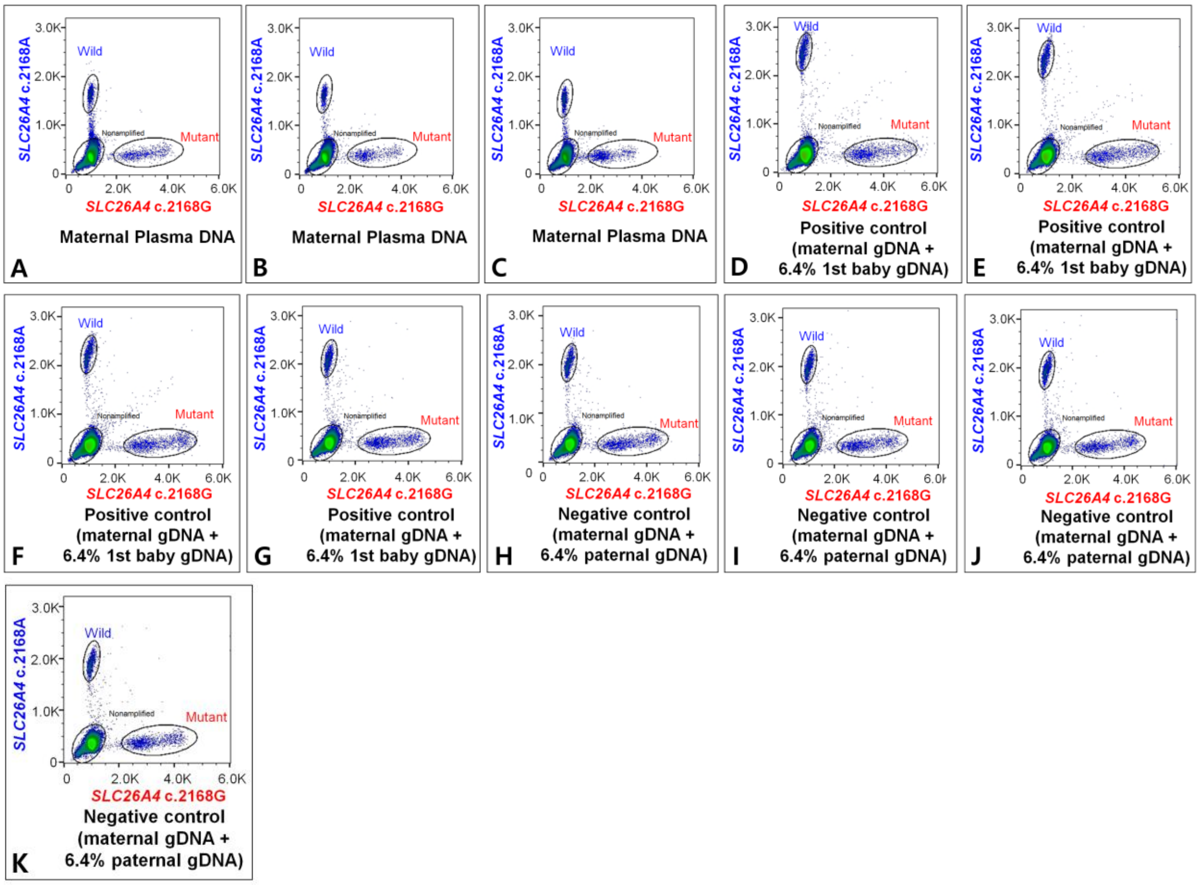


**Supplementary Figure S3.** Two-dimensional histogram of the maternal mutation (*SLC26A4* c.2168A>G (p.H723R)) in maternal plasma DNA (A-C) and positive (D-G) and negative (H-K) control of the first family.

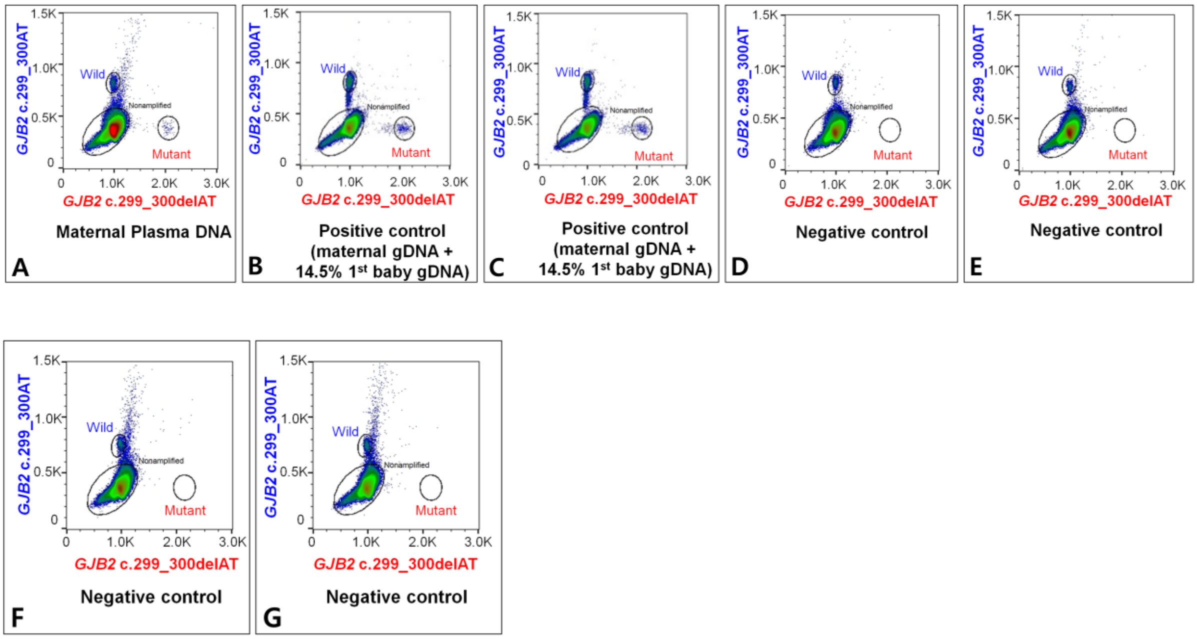


**Supplementary Figure S4.** Two-dimensional histogram of the paternal mutation (*GJB2* c.299_300delAT (p.H100Rfs*14)) in maternal plasma DNA (A) and positive (B, C) and negative (D-G) control of the second family.

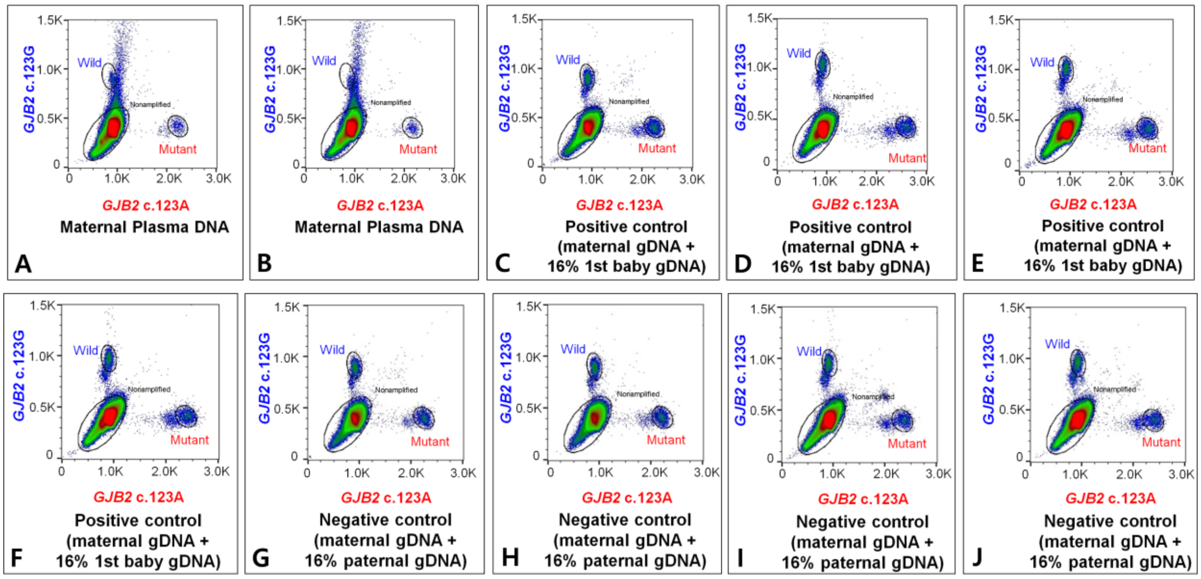


**Supplementary Figure S5.** Two-dimensional histogram of the maternal mutation (*GJB2* c.123G>A (p.G45E)) in maternal plasma DNA (A, B) and positive (C-F) and negative (G-J) control of the second family.

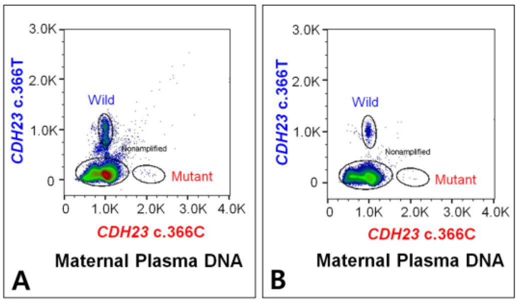


**Supplementary Figure S6.** Two-dimensional histogram of SNP exclusively for the father (*CDH* c.366C>T (p.V122V)) in maternal plasma DNA (A, B) of the third family.

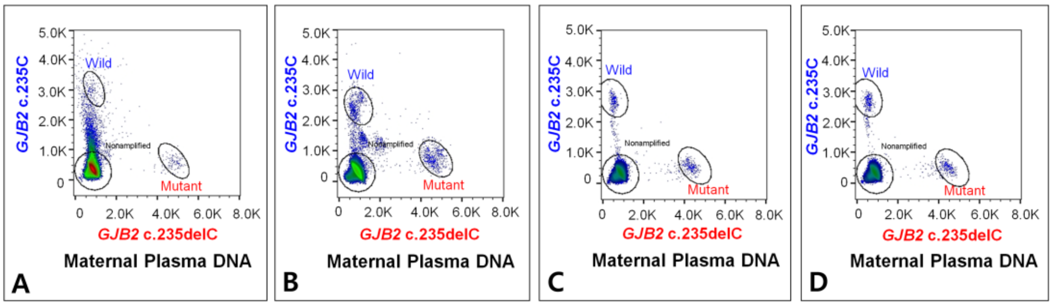


**Supplementary Figure S7.** Two-dimensional histogram of the paternal and maternal mutation (*GJB2* c.235delC) in maternal plasma DNA (A-D) of the third family.

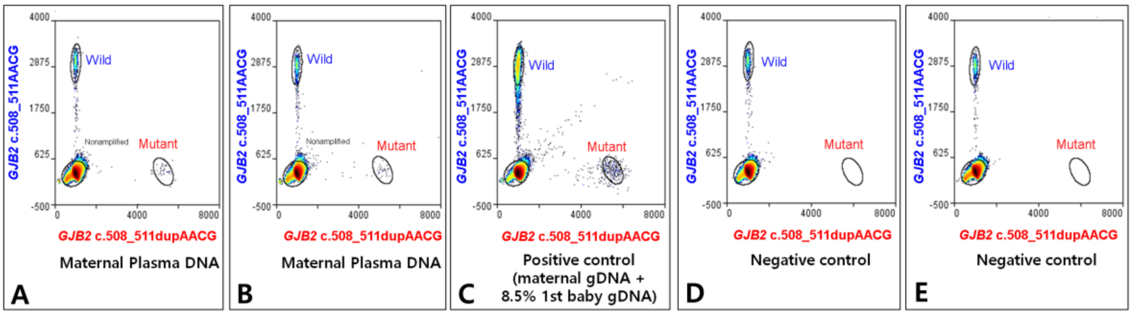


**Supplementary Figure S8.** Two-dimensional histogram of the paternal mutation (*GJB2* c.508_511dupAACG (p.A171Efs*40)) in maternal plasma DNA (A, B) and positive (C) and negative (D-E) control of the fourth family.

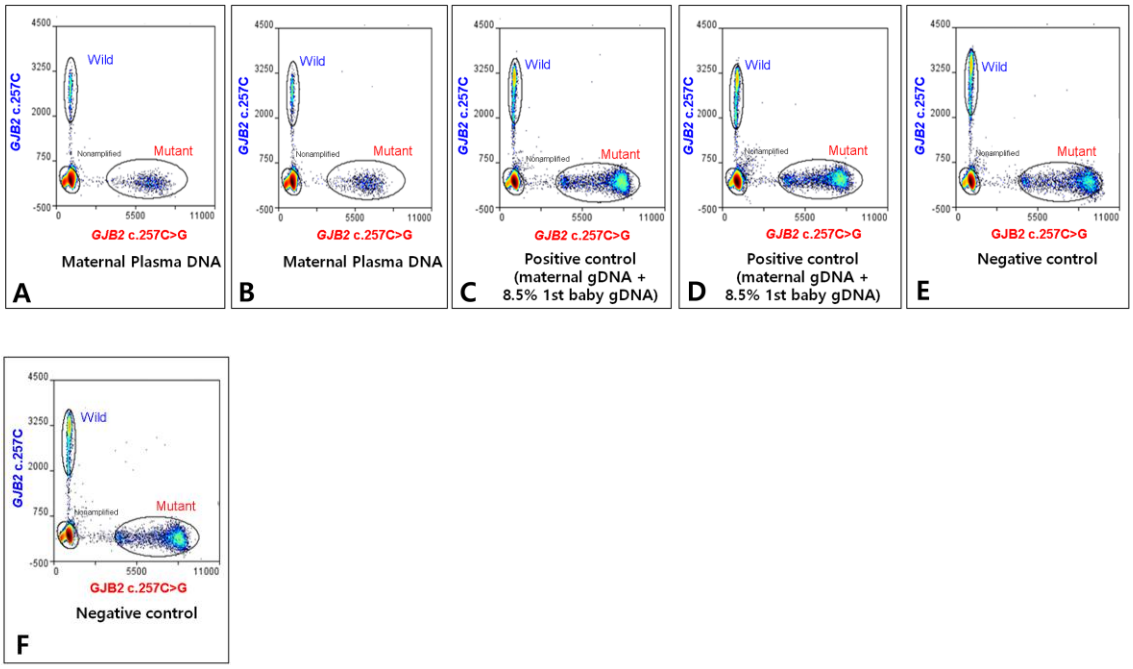


**Supplementary Figure S9.** Two-dimensional histogram of the maternal mutation (*GJB2* c.257C>G (p.T86R)) in maternal plasma DNA (A, B) and positive (C, D) and negative (E, G) control of the fourth family.
